# Artificial Intelligence in Medical Imaging and Its Application in Sonography for the Management of Liver Tumor

**DOI:** 10.3389/fonc.2020.594580

**Published:** 2020-12-21

**Authors:** Naoshi Nishida, Masatoshi Kudo

**Affiliations:** Department of Gastroenterology and Hepatology, Kindai University Faculty of Medicine, Osaka-Sayama, Japan

**Keywords:** artificial intelligence, ultrasound, imaging, liver cancer, neural network, diagnosis

## Abstract

Recent advancement in artificial intelligence (AI) facilitate the development of AI-powered medical imaging including ultrasonography (US). However, overlooking or misdiagnosis of malignant lesions may result in serious consequences; the introduction of AI to the imaging modalities may be an ideal solution to prevent human error. For the development of AI for medical imaging, it is necessary to understand the characteristics of modalities on the context of task setting, required data sets, suitable AI algorism, and expected performance with clinical impact. Regarding the AI-aided US diagnosis, several attempts have been made to construct an image database and develop an AI-aided diagnosis system in the field of oncology. Regarding the diagnosis of liver tumors using US images, 4- or 5-class classifications, including the discrimination of hepatocellular carcinoma (HCC), metastatic tumors, hemangiomas, liver cysts, and focal nodular hyperplasia, have been reported using AI. Combination of radiomic approach with AI is also becoming a powerful tool for predicting the outcome in patients with HCC after treatment, indicating the potential of AI for applying personalized medical care. However, US images show high heterogeneity because of differences in conditions during the examination, and a variety of imaging parameters may affect the quality of images; such conditions may hamper the development of US-based AI. In this review, we summarized the development of AI in medical images with challenges to task setting, data curation, and focus on the application of AI for the managements of liver tumor, especially for US diagnosis.

## Introduction

Artificial intelligence (AI) is generally considered as the intelligence performed by compactional statistics, where machine learning is a subset of AI. Recently, AI is emerging as a major constituent in the field of medicine and healthcare. In particular, AI can be easily applied to imaging data because these data are electronically organized, and AI excels at recognizing unique and complex features of images and facilitates quantitative assessments in an automated fashion. This characteristic of AI is ideal in the constrained clinical setting wherein medical staff must interpret large image datasets based on their visual perception with uncertainty, in which human errors are inevitable. For example, AI is a powerful tool in radiomics where extracting a large number of features form medical images is required. Based on this advantage, AI have been applied for classification of lesions, such as liver tumors, and prediction of the prognosis using image data from computed tomography and magnetic resonance imaging (MRI) (1). In addition, AI-based image processing techniques have also introduced in the field of ultrasonography (US). This review shows the recent progress in AI for medical imaging, especially for an AI-aided diagnosis for the detection, characterization, subsequent monitoring, and prediction of outcomes in patients with liver cancer, especially in the field of US diagnosis.

## History and Recent Progress of AI in Medical Imaging

The application of pattern recognition in medical issues has been proposed in the early 1960s. In the 1980s, the prevalence of computers induced the development of medical AI in radiology using a quantitatively computable domain. After the emergence of deep neural network, the rate at which AI is evolving radiology is rapidly growing that is proportional to the growth of data volume in medical image and computational power (2).

For the image analysis, a convolutional neural network (CNN) is commonly applied, which is a class of deep neural networks using pixel value and assembling complex patterns to smaller and simpler patterns (2). The algorism contains multiped hidden layer with multiple convolutional and pooling layers. A trained CNN-based AI model using ≥120,000 retinal fundus images has been demonstrated to show high performance comparable to that of an experienced ophthalmologist for detecting referable diabetic retinopathy, which is expected to effectively assist ophthalmologists in the clinical workflow (3). Assessment of AI models for detecting lymph node metastasis of breast cancer based on whole microscopic slide images showed the superior performance of AI for detecting cancer cells in specimens to that of pathologists (4). A pre-trained CNN-based AI model for the diagnosis of skin cancer achieves performance on par with that by expert dermatologists in terms of the discrimination of skin cancers from corresponding benign lesions on dermography (5). AI models for the detection of pediatric pneumonia on chest radiography images and for the discrimination of diabetic macular edema from age-related macular degeneration on optical coherence tomography images are also reported with high performance, comparable to that of human experts (6). An AI-based colonoscopy system has been shown to accurately differentiate neoplastic lesions from non-neoplastic lesions on stained endocytoscopic images and endocytoscopic narrow-band images in endoscopic evaluation of small colon polyps (7). The application of AI for US-based diagnosis has been mainly reported for the diagnosis of malignant tumors, such as mammary and thyroid cancers (8–11). Le et al. reported an AI model for the diagnosis of thyroid cancer pre-trained with 312,399 B-mode US images of cancer and healthy controls (12). The model’s diagnostic performance was validated in three test datasets with AUCs of 0.908–0.947. The AI model showed higher specificity in identifying thyroid cancer and comparable sensitivity to those corresponding to experienced radiologists. Another report described a real-time detection system of thyroid tumors based on real-time images using the “You Only Look Once” (YOLO) algorithm. This model achieved a similar sensitivity, positive predictive value, negative predictive value, and accuracy for the diagnosis of malignant thyroid tumors with higher specificity compared to those corresponding to experienced radiologists (12, 13). For the detection of breast cancer, Kumar et al. reported a real-time segmentation model of breast tumors using a CNN (14). This system can reportedly segment tumor images in real-time, suggesting its potential for clinical applications. Collectively, diagnostic accuracy of well-trained AI model for medical image is, at least, on par with human experts with much quicker output, suggesting the higher efficiency for diagnosis in clinical setting.

On the other hand, recently, Skrede et al. reported the use of AI for the prediction of outcomes after colorectal cancer resection using a pre-trained CNN-based model with pathological images (15). They discriminated the cases of poor prognosis from those of good prognosis, indicating the potential of medical AI for the management of cancer, such as the identification of patients who would benefit from adjuvant treatment after resection.

## Process for Developing AI Models for Imaging Diagnosis

### Setting Tasks for AI in Medical Imaging

For the development of AI in medical imaging, it is important to select tasks that reflect important needs at clinical sites. For example, large-volume screening of medical images requires extensive effort, which is time consuming and invites human errors. In this setting, AI should be a powerful tool for clinicians because of its advantageous for precise detection of subtle features of lesions, segmentation, and quick output. AI models that can estimate the risk of disease may contribute to avoiding invasive examinations, representing an attractive task (16).

### Data Sets for Developing AI Models for Medical Images

Generally, three independent datasets are required for developing medical AI (17). A training set is required for the training of AI models, which contains many images to update model parameters. A tuning set is for the selection of a model’s hyperparameters that are necessary for the best expected output. A test set is for the final assessment of the performance of AI models. The splitting of curated data must be clean, and each dataset should be completely independent without any overlap with respect to lesions to avoid overfitting the output.

For disease classification, such as that corresponding to diagnosis, the data volume in each subclass should be similar because imbalances in data volumes among subclasses may lead to overfitting of the output, which may limit the performance of an AI model. For the image of rare diseases, the AI-based image created through generative adversarial networks might also be applicable.

### AI Algorithm

During training, AI models automatically detect specific features of images through the fitting of model parameters, which improves the performance. CNNs are commonly applied for AI algorithm of imaging data (2). However, US examinations require real-time output, and an algorithm that requires many mathematical operations might not be appropriate for analyzing US images. The YOLO-based algorithm is suitable for the real-time detection and classification of lesions with high-speed processing. The process of selecting a model’s architecture and training essentially involves a balance between model underfitting and overfitting (17). Underfitting occurs when a low-capacity model is used relative to the problem complexity and data size. Overfitting indicates that the evaluation overestimates the model’s performance on previously unencountered data, in which case low performance on the test set is observed. Because there is a large diversity among US images in terms of the conditions of the examination and image parameter settings, larger volumes of data are required compared to those required for the development of other medical imaging AI.

### Evaluation of Performance and Potential Impact

One of the major categories of evaluation of AI-aided imaging diagnosis is the ability to discriminate the lesions, such as benign or malignant. The area under the receiver operating characteristic curve (AUC) is commonly used as a threshold-free discriminative metric. Evaluation may also be based on other metrics, such as sensitivity (recall), specificity, and precision (positive predictive value); these are threshold-dependent. On the other hand, calibration, which evaluates how effectively the predicted probability matches the actual diagnosis should also be estimated (17). In addition, variability in the probability in the same lesion may also need to be analyzed because there can be variations among US images even within the same lesion, which is attributed to differences in parameter settings. Validation for accuracy is a critical process in the transitional process of medical AI. The performance of AI models must be evaluated using independent test cohorts and be compared with an experienced human control in real-world scenarios.

## Current AI Models for Medical Imaging of Liver Lesions

### AI Using Medical Image for the Management of Liver Tumors

Recently, many reports have described the development of AI models for the detection and diagnosis of liver tumors; some studies have aimed to predict outcomes after treatments, which may be applicable for the personalized management of patients (18, 19).

Hamm et al. reported the classification of 6 types of liver tumors by a pre-trained CNN using MRI data of 494 lesions from 334 cases (20). After data augmentation of the images for training, the established AI model demonstrated 90% sensitivity and 98% specificity for the test cohort. The average sensitivity and specificity for the radiologist were 82.5 and 96.5%, respectively. For the diagnosis of hepatocellular carcinoma (HCC), the sensitivities were 90% for the AI model and 60–70% for the radiologists. Considering the short processing time (only 6.6 ms) for output, the pre-trained AI model showed superior performance compared to that of the human radiologists.

On the other hand, AI is also useful for the detection of specific radiological features that may reflect histopathological characteristics associated with the biological behavior of a tumor. From this point of view, the development of AI for the prediction of outcomes after treatment, including tumor recurrence after surgery, may be possible. If pathological diagnosis is applied for constructing an AI model for medical imaging, it may be a non-invasive substitute for biopsy, which may significantly impact the management of cancer. Fent et al. reported a preoperative prediction model for microvascular invasion in patients with resectable HCC who do not show macroscopic vascular invasion through training using gadolinium-ethoxybenzyl (EOB)-diethylenetriamine-enhanced MRI data (21). The AI model selected ten specific features of EOB-enhanced MRI data to predict microvascular invasion. The performance of the AI model showed an AUC of 0.83 with 90.0, 75.0, and 84.0% sensitivity, specificity, and accuracy, respectively, which were much better than those of human radiologists. Kim et al. reported an AI model for the prediction of early and late recurrence of tumors after surgery using EOB-MRI data from solitary HCC cases (22). They established their AI model using a random survival forest to predict disease-free survival and found that peritumoral image features 3 mm outside the tumor border are important for the prediction of early recurrence after curative surgery.

### AI Using Histopathological Images for Diagnosis and Management of Liver Cancers

It has also been reported that an AI model pre-trained with histopathological images of liver cancer using transfer learning can distinguish cancerous tissue from healthy liver tissue (23). Saillard et al. showed that a deep-learning model of histopathological images predicts survival after resection of HCC (24). They developed two kinds of AI models pre-trained with supervised image data, which was annotated based on the tumor portion in the slide images by pathologists, and non-supervised data without human annotations. The concordance indices for survival prediction were 0.78 and 0.75 for the pre-trained AI models with supervised and non-supervised data, respectively. Reportedly, these histopathological AI models showed a higher discriminatory power than that derived from a combination of known clinical risk factors. Some pathological findings, including vascular space, a macrotrabecular pattern of tumor cell architecture, a high degree of cytological atypia, and nuclear hyperchromasia, effectively predicted poor survival, and immune infiltrates and fibrosis in tumor and non-tumor tissues were associated with a low risk of short survival. These studies indicate that histopathological images yield useful training data for the prediction of prognosis in HCC cases (25).

### AI-Aided Diagnosis for Liver Tumors in Ultrasonography

Generally, US images are heterogeneous because of the multiple image parameters and conditions of examination compared to other kind of medical images. Such heterogeneity of image data makes it difficult to develop the AI for US diagnosis, especially for liver tumors (18).

AI models are trained using cropped images of regions of interest that specifically focus on tumors for applying neural network and can be evaluated using cross-validation methods for small sample cohorts. The studies regarding the application of B-mode US images on machine learning for the diagnosis of liver tumor are summarized in **Table 1**. Virmani et al. reported the machine learning for discriminating HCC and metastatic liver tumor using support vector machine (SMV), where overall accuracy was 91.6 %; sensitivity of 90% for HCC and 93.3% for metastatic tumor were achieved (26). Hwang et al. tried to extract textural features of liver tumors including cysts, hemangiomas, and malignant lesions for the diagnosis; they examined the accuracy of two-class discriminations for cyst vs. hemangioma, cyst vs. malignant tumor, and hemangioma vs. malignant tumor, demonstrating the accuracy of more than 95% for each comparison (28). On the other hand, the study using artificial neural network (ANN) show 4-class discrimination for normal liver, cyst, hemangioma, and HCC: accuracy of almost 90%, and similar levels of sensitivity, and specificity are reported (29). Generally, these early studies failed to show the superiority of neural network for the diagnostic accuracy of liver tumors compared to the conventional machine learning because of the small size of learning cohort. Schmauch et al. reported the performance of an AI model for the diagnosis of liver tumors from B-mode US images (30). They reported an AI model for lesion detection and diagnosis from whole-liver US images using a 50-layer residual network. Despite the relatively small volume of training data, the performance for tumor detection and 5-class discrimination (HCC, metastatic tumors, hemangiomas, cysts, and focal nodular hyperplasia) achieved considerable AUCs (0.953 and 0.916) for tumor detection and discrimination, respectively, by cross validation. To reduce the heterogeneity of the US images, they cropped the images maximally to remove the black borders and standardize the aspect ratio. They also performed rescaling of the image intensity for normalization based on the intensity of the abdominal wall.

**Table 1:** Performance for diagnosis of liver tumor based on the machine learning using image of ultrasonography.

Algorism	Liver lesions:number of the cases	Performance	References
Pre-trained AI using B-mode US images			
SVM	normal liver: 15 cirrhotic liver: 16 HCC: 25	accuracy: 88.8%	(26, 27)
SVM	HCC: 27 metastatic tumor: 27	overall accuracy: 91.6% sensitivity: 90% for HCC 93.3% for metastatic carcinoma	(26)
ANN	cyst; 29 hemangioma: 37 malignant tumor: 33	cyst vs. hemangioma accuracy: 99.7% cyst vs. malignant accuracy: 98.7% hemangioma vs. malignant accuracy: 96.1%	(28)
ANN (sparse autoencoder)	normal liver: 16 cyst: 44 hemangioma: 18 HCC: 30	accuracy: 90.5% sensitivity: 91.6% specificity: 88.5%	(29)
CNN	non-tumorus liver: 258 hemangioma: 17 metastatic tumor: 48 HCC: 6 cyst: 30 focal nodular hyperplasia: 8	AUC for tumor detection: 0.935 AUC for tumor discrimination: 0.916 (mean)	(30)
Pre-trained AI using CEUS images			
ANN	hemangioma: 16 focal fatty liver: 23 HCC: 41 metastatic tumor: 32 hypervascular: 20 hypovascular: 12	accuracy: 94.5% sensitivity: 93.2% specificity: 89.7%	(31)
SVM	benign tumor: 30 malignant tumor: 22	accuracy: 90.3% sensitivity: 93.1% specificity: 86.9%	(32)
SVM	benign tumor, HCC, or metastatic tumor 98	benign vs. malignant accuracy: 91.8% sensitivity: 94.0% specificity: 87.1% benign vs. HCC vs. metastatic carcinoma accuracy: 85.7% sensitivity: 84.4% specificity: 87.7%	(33)
SVM (multiple kernel learning)	benign tumor: 46 malignant tumor: 47	accuracy: 90.4% sensitivity: 93.6% specificity: 86.9%	(34)
Logistic regression analyses	Solitary HCC without macrovascular invasion: 468	Prediction of microvascular invasion using ultrasomics feature: AUC = 0.731	(35)
SVM	HCC: 235 High-grade: 65 Low-grade: 170	Discrimination of HCC pathological grades using ultrasomics and clinical factors: AUC = 0.785	(36)
CNN	HCC before TACE: 130	Prediction of response to TACE: AUC = 0.93	(37)
CNN	HCC: 419 patients who underwent RFA; 214 patients who underwent resection; 215	prediction of RFS for 2 years after curative treatment C-index 0.726 for RFA C-index 0.726 for resection	(38)

SVM, support vector machine; ANN, artificial neural network; CNN, convolutional neural network; CEUS, contrast-enhanced ultrasonography; HCC, hepatocellular carcinoma; AUC, area under the receiver operating characteristic curve; TACE, transarterial chemoembolization; RFA, radiofrequency ablation; C-index, concordance index.

In addition to the gray scale B-mode US, doppler US, contrast-enhanced US (CEUS), shear wave elastography (SWE) and three-dimensional US images are also applicable for the training of AI models. Still image of contrast-enhanced US (CEUS) was applied for the learning data for more accurate discrimination of liver tumors. Streba et al. applied ANN for 4-class discrimination of liver tumor with 94.5, 94.2, and 89.7% for accuracy, sensitivity and specificity, respectively, for the discriminaton (31). Gatos et al. and Kondo et al. reported the 4-class classification of benign tumors, hepatocellular carcinoma, and metastatic tumors using SMV pretrained with CEUS images (32, 33). A contrast agent, Sonazoid, was used and, reportedly, sensitivity, specificity, and accuracy that discriminate malignant lesions from benign were 94.0, 87.1, and 91.8%, respectively (33). Another report applied a pretrained SMV using CEUS images and achieved the accuracy, sensitivity, and specificity of 90.4, 93.6, and 86.9%, respectively, for the different diagnosis of benign and malignant liver tumors (34). Discrimination of benign and malignant lesions is a critical task for the management of patients with liver tumors, and CEUS images yield attractive data for the development of AI models to detect malignant tumors.

On the other hand, because of the development of new treatments in HCC, management of this type of cancer is becoming complex (39). Recently, in addition to detection and diagnosis, AI model regarding the management of HCC, such as prediction of microvascular invasion, pathological grading, and treatment outcomes have been reported. Hu et al. proposed US-based radiomics score consisted of six selected features was an independent predictor of microvascular invasion in HCC (35). On the other hand, model for predicting pathological grading of HCC before surgery was also reported using ultrasomics of CEUS images (36). Liu et al. developed an AI model for the prediction of responses to transarterial chemoembolization in patients with HCC through training with B-mode US and CEUS images (37). They reported AUCs of 0.93 and 0.81 for the AI based on CEUS and B-mode US images, respectively, indicating a higher performance of the model pre-trained with CEUS images than that with B-mode US images. They also reported AI models for predicting outcomes in patients with HCC after two types of treatment—radiofrequency ablation (RFA) and liver resection—from radiomics information based on CEUS images (38). For the prediction of two-year progression-free survival (PFS), both models provided high prediction accuracy. Interestingly, the models showed that some patients who underwent RFA and surgery should swap their treatments, so that a higher probability of increased 2-year PFS would be achieved. In addition, another report showed radiomic signature from grayscale US images of gross-tumoral region had potential for prediction of microvascular invasion of HCC before surgery, suggesting the potential of radiomic approach for the prediction of outcome (40). Such AI prediction models using radiomic signature may be applicable for personalized medicine in HCC treatment.

The grading of liver fibrosis and steatosis is also an important task for the management of liver disease because these backgrounds may confer a risk of liver cancer. Several reports have described the classification of fibrosis and steatosis based on disease progression using AI models trained with B-mode US and SWE images (18, 41). Deep-learning models show hyper-performance in terms of detection and risk stratification of fatty liver disease compared to that corresponding to conventional machine-learning models (42). AI models pre-trained with color images of US-SWE can also discriminate chronic liver disease from healthy cases (43). Reportedly, the combination of B-mode US images, raw radiofrequency data, and dynamic contrast-enhanced microflow is a useful dataset for developing AI models that classify the stage of liver fibrosis (44), where datasets involving raw radiofrequency data provide better predictive value than those from conventional US image only. Therefore, it should be possible that AI using multiparametric ultrasomics can help improve the performance of the model. For the development of AI that determine the stage of liver fibrosis more accurately, Gatos et al. reported a detection algorithm that excludes unreliable regions on SWE images, which contributes to a reduction in interobserver variability (45). Applying these AI models may be an alternative to invasive liver biopsy for predicting the progression of liver disease, which may be associated with a risk of liver cancer.

## Conclusion

Among the imaging modalities, US is the most commonly used in clinical practice for detection of liver tumors because of its low-cost, non-ionizing, and portable point-of-care characteristics providing real-time images. From this point of view, the AI-powered US carries more advantage in routine clinical applications compared to that in CT and MRI (46). Although, US images involve operator-, patient-, and scanner-dependent variations, AI-aided US diagnosis is becoming mature that is attributed to the recent advancement in the US equipment and increase in computing power to identify the complex imaging features. In addition to the B-mode image, images from CEUS and US elastography is becoming promising data applicable in AI-based diagnosis in the field of liver tumor according to the prevalence of high-end US equipment (46, 47). These could also be a safeguard for misdiagnosis in the actual workflow. The development of AI-aided technologies for the detection and diagnosis of malignant tumors may carry sufficient potential to reduce cancer-related mortality in the near future.

## Author Contributions

Conceptualization: NN. Writing—original draft preparation: NN. Writing—review and editing: NN. Supervision: MK. Funding acquisition: NN and MK. All authors contributed to the article and approved the submitted version.

## Funding

This work was supported by AMED (Japan Agency for Medical Research and Development) under Grant No. JP19lk1010035. (NN and MK) and ROIS NII Open Collaborative Research 2020 under Grant No. 20S0601 (NN).

## Conflict of Interest

The authors declare that the research was conducted in the absence of any commercial or financial relationships that could be construed as a potential conflict of interest.
